# Neurotoxic symptoms among anesthesia technicians in operating rooms

**DOI:** 10.1192/j.eurpsy.2026.11526

**Published:** 2026-07-31

**Authors:** G. Mohamed Anis, S. Imen, H. Aicha, F. Afef, H. Asma, M. Mohamed Larbi, H. Mounira, H. J. Kaouthar

**Affiliations:** 1 Occupational Medicine; 2 Rheumatology Department, University Hospital Hedi Chaker; 3 Physiology and Functional Explorations, University Hospital Habib Bourguiba, Sfax, Tunisia

## Abstract

**Introduction:**

The operating room environment exposes healthcare professionals to multiple chemicals. Few studies addressed the health effect of this chronic exposure, which is often insidious and underestimated, particularly in terms of neurotoxic effects.

**Objectives:**

The aim of this study was to assess signs of neurotoxicity among anesthetic technicians (AT) and evaluate its associated factors.

**Methods:**

We conducted a cross-sectional study among AT in two University Hospitals in Sfax, Tunisia, between January and October 2024. Sociodemographic and professional data were collected. Physical exertion was evaluated on a scale of 1 to 10. A screening of symptoms of neurotoxicity was done using the Swedish Questionnaire 16 (Q16).

**Results:**

Our population consisted of 77 AT with a mean age of 47.32±7.74 years. Five participants (6.5%) were males. The median Body Mass Index (BMI) of our population was 25.71 IQR [23.87; 28.94]. Medical history was noted in 93.5% of the population. Psychiatric and neurological diseases were found in 15.6% and 9.1% of the population respectively. Physical and Leisure activities were found in 13% and 24.7% of the population respectively. The median job tenure was 7 years IQR [3; 13]. The median of the physical exertion was 7 IQR [5; 8]. Exposure to inhalational anesthetic agents was reported by 88% of AT. The median Q16 score was 8 IQR [5; 9]. An abnormal score was found in 72.7% of the population. Multivariate analysis showed that Medical History (β=0.256; p=0.009), current job tenure (β=0.275; p=0.034) and exposure to inhalational anesthetics (β=0.261; p=0.007) were significantly associated with a higher Q16 score. Male sex (β=-0.323; p=0.001), Leisure activity (β=-0.354; p<0.001) and working in HC (β=-0.300; p=0.002) were associated with a lower Q16 score.

**Conclusions:**

Our results showed alarming neurotoxic symptoms among AT that need to be confirmed through specialized medical investigations. Moreover, longitudinal studies are needed to establish the casual link between these symptoms and the occupational factors that have emerged in our multivariate analysis.

**Disclosure of Interest:**

None Declared

